# Causes of severe injuries in wheelchair user motor vehicle passengers: pilot study for future safety restraint system

**DOI:** 10.1265/ehpm.26-00037

**Published:** 2026-05-27

**Authors:** Masahito Hitosugi, Ayumu Kuwahara, Yoshitake Kitaguchi, Katsushi Yoshii, Masaki Inoue, Marin Takaso, Mami Nakamura, Nick Barua

**Affiliations:** 1 Department of Legal Medicine, Shiga University of Medical Science, Tsukinowa, Seta, Otsu, Shiga 520-2192, Japan; 2 Joyson Safety System Japan

**Keywords:** Wheelchair, Vehicle passenger, Aged person, Seatbelt, Restraint system, Motor vehicle collision, Safety

## Abstract

**Background:**

As population aging progresses in Japan, an increasing number of older wheelchair users regularly travel in the rear space of motor vehicles. Poor seatbelt fit is the main cause of severe or fatal injuries in collisions. The current study sought to examine the potential risks regarding seatbelts for wheelchair users in Japan using national statistics, and to elucidate the mechanisms of injuries among wheelchair users involved in frontal collisions using sled tests. We propose a restraint system for wheelchair users traveling in vehicles.

**Methods:**

To identify cases of collisions involving vehicles with wheelchair user passengers, national police database was examined. A sled test representing a 48 km/h frontal collision with a Hybrid III AF05 dummy was used to examine kinematics at collision and biomechanical parameters of wheelchair users. We examined three test conditions (1. generally used seatbelt path for standard wheelchairs; 2. close seatbelt path for standard wheelchair; 3. close seatbelt path for sturdy wheelchair).

**Results:**

According to a national database in Japan, a seatbelt was worn in most cases of fatalities and casualties among wheelchair user passengers. In sled test condition 1, the dummy moved forward and the lap belt compressed the abdomen. High values were observed for head injury criterion and neck injury criterion. In condition 2, the dummy moved forward and fell because the wheelchair seat collapsed. In all conditions, high values for chest deflections (51.3 to 53.8 mm) were caused by high shoulder-belt load (8,217 to 8,660 N).

**Conclusions:**

Indications for future design of safety restraint system were provided as follows: both the shoulder and lap belts would be correctly positioned across the bony parts of the shoulder and pelvis, respectively; a force limiter would be installed in the restraint system to reduce shoulder belt loads; a sturdy wheelchair and adequate tie-down system that can resist applied forces would be used.

## Background

In Japan, the proportion of older people in the population is increasing. People aged 65 years or more accounted for 29.1% of the total population in 2023. Population aging causes an increased number of older wheelchair users. Older people often receive care services (e.g., day care with rehabilitation) requiring transfer from their homes to care facilities via motor vehicle. Transportation often requires wheelchair users to board the vehicle from the rear. Recently, an increasing number of wheelchair user vehicle passengers have been involved in fatal motor vehicle collisions (MVCs). We reviewed all fatal MVCs in Shiga Prefecture, Japan, which has a population of approximately 1.4 million people, from 2017 to 2022. The rate of wheelchair users in fatal collisions involving motor vehicle passengers increased from 3.6% in 2017 to 2019 to 7.8% in 2020 to 2022 [1]. As population aging progresses in developed countries, fatalities caused by this type of collision may increase in future. We previously performed a deep analysis of these fatalities, revealing that most other passengers in the vehicles involved in these collisions suffered from minor injuries or no injuries [2]. Additionally, the results indicated that some fatal injuries were caused by inappropriate seatbelt restrictions [2]. Lap and shoulder seatbelts do not adequately fit most wheelchair user passengers because of the way wheelchairs are constructed. Because lap belts pass over or under wheelchair handrails, they do not adequately fit the lumbar region of wheelchair users. Therefore, the movement of the body immediately after the collision can cause the wheelchair user passengers’ body to collide with the interior of the vehicle, or sustain damage from the intrusion of the lap belt into the abdomen. The passenger may subsequently suffer from severe head, chest or abdominal injuries. To prevent these injuries, it is necessary to identify the contributing factors. Dynamic testing using a sled device and a dummy model can be useful for simulating real-world vehicle collisions and quantitatively estimating the forces applied to the body of the dummy. In previous studies, sled tests were performed to examine the impact on a wheelchair user model in a vehicle [3–5]. However, no previous studies have examined collisions in which wheelchair users are located in the rear space of the vehicle, with seatbelt paths. Furthermore, alternative safety systems for preventing such fatalities have not been examined.

In the current study, we first examined the potential risks regarding seatbelts for wheelchair users from a national database of MVC statistics in Japan. Second, we sought to elucidate the mechanisms of injuries of wheelchair users involved in a frontal collision with sled tests. Although this is the pilot study combining descriptive accident data analysis with biomechanical experimentation, we propose a restraint system for wheelchair users boarding a vehicle.

## Methods

### Trends in vehicle collisions involving wheelchair user passengers

The Institute for Traffic Accident Research and Data Analysis (ITARDA) in Japan maintains a large, all-inclusive database of MVCs using data provided by Japan’s National Police Agency (NPA). We used this database to identify cases of collisions involving vehicles with wheelchair user passengers. Although the term “wheelchair user passengers” was not used for searching the cases in this database, we collected cases with the terms “wheelchair transport vehicle” and “rear passenger.” Therefore, these categories encompassed vehicle collisions in which the wheelchair user was located in the rear space of the vehicle. However, because not all vehicles that transport wheelchair users are registered as “wheelchair transport vehicles,” the collected cases would not be expected to constitute all collisions involving wheelchair user passengers throughout Japan.

In each case, we examined the severity of injuries. Wheelchair users’ injury levels were categorized as death, severe injury, or mild injury, on the basis of physicians’ diagnoses. Death was defined as a fatality occurring within 24 hours of the collision. Cases in which a wheelchair user suffering from injuries required treatment for 30 days or more were defined as severe injury. Cases in which treatment was required for less than 30 days were defined as mild injury.

Seatbelt use was examined, and classified as “used,” “not used,” or “unknown.”

### Sled test using a dummy model

#### Test setup

To examine the kinematics and biomechanical parameters of wheelchair users in a collision, sled tests simulating a frontal collision were performed. The procedure of the sled test complied with the International Organization for Standardization (ISO) standards 10524-1 and 7176-9, which define the safety of the wheelchair boarding on a vehicle. Additionally, the procedure complied with the safety certification test defined by the United States Federal Motor Vehicle Safety Standards (FMVSS). The sled test used a collision velocity of 48 km/h with a 20 gravitational acceleration (G) pulse. Trapezoid waveforms measured in a flat barrier test with vector velocity changes at the time of impact of 48 km/h were applied to the sled. The situation was simulated as a real-world collision involving a wheelchair user riding in the rear space of the wheelchair transport vehicle using a seatbelt. The dummy used in the present study was the Hybrid III AF05 (representing a person in the 5^th^ percentile for body weight relative to the average American female). This dummy has a similar stature to that of an average older Japanese person with a height of 153 cm, and has been confirmed for use in official crash tests.

#### Restraint condition

**Fig. 1:**
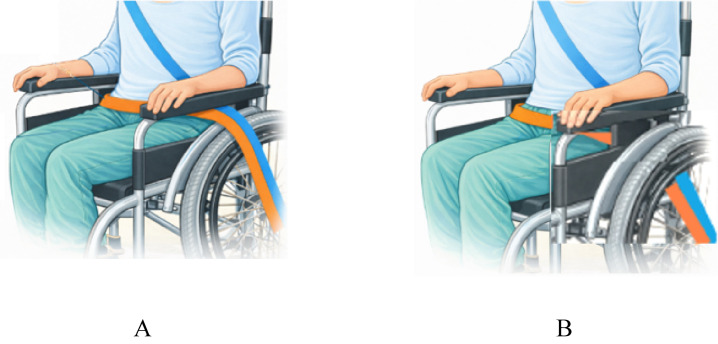
The seatbelt path of the dummy. The seatbelt path of the dummy is shown (A: Test 1, B: Test 2 and 3).

Lap and shoulder belts were used for testing in the current study, as found in most vehicles with wheelchair passengers. The anchorage point of the shoulder belt was determined by simulating currently available wheelchair transporting vehicles in which shoulder belts are correctly positioned on wheelchair user passengers. As the center of the intersection of the seat and backrest was the reference position, the anchorage point of the shoulder belt was determined as 240 mm posterior, 380 mm to the left and 632 mm above; the position of the buckle of the shoulder belt was determined as 9 mm posterior, 390 mm to the right and 363 mm below. The anchorage point of the lap belt was determined as 10 mm forward, 390 mm to the right and 293 mm below the reference position; the position of the buckle of the lap belt was determined as 3 mm forward, 460 mm to the left and 373 mm below the reference position. These positions corresponded to UN Regulation No. 14, which contains uniform provisions concerning the approval of vehicles with regard to safety-belt anchorage defined by the United Nations. Force limiter was not activated in all tests. In test 1, the shoulder belt passed the right clavicle, the center of the chest of the dummy, between the left armrest and the side board, then connected to the buckle located on the floor. The lap belt passed between the arm rest and the side board of both sides (Fig. 1-A). In test 2, both belts passed between the spokes of the wheels and tried to pass near the lumbar region of the dummy. In tests 1 and 2, we used a standard wheelchair (Esluve Wheelchair, Tokyo Jitsugyo, Tokyo) in each case. In test 3, we used a sturdy wheelchair that was resistant to forces of 20 G (MZ-1, Matsunaga Corporation, Co, Ltd, Gifu) with a similar seatbelt path to that in test 2 (Fig. 1-B).

#### Measurements

The following items were obtained from the test, and biomechanical parameters were subsequently calculated.

1) Kinematics of the dummy
  The overall kinematics of the dummy, including the trajectory during impact, were measured using a high-speed (1,000 fps) video recorder (GX-1, nac Image Technology Inc, Tokyo).
2) Acceleration, displacement, and moment
  Accelerations of the head, chest and the pelvis along the horizontal (X), vertical (Y), and depth (Z) axes were measured by the accelerometer installed in the dummy. The resultant accelerations of the head, chest, and pelvis were calculated from the square root of the sum of the squares of their respective x, y, z accelerations. Displacements of the head, neck, chest, pelvis and deflection of the chest along the horizontal direction were measured. Displacements of the head, chest, and pelvis were calculated by integrating the respective X-axis accelerations twice. In addition, the moment around the Y-axis of the neck was measured, corresponding to extension or flexion of the neck. The loads on the shoulder belt were measured using load cells.
  Data were digitized with a high-speed data acquisition system with a sampling rate of 20 kHz, then filtered with a channel frequency class 180 filter (chest and pelvis data) or a class 1,000 filter (head and neck data).
3) Injury criteria
  First, the head injury criterion (HIC) was calculated. This version of the HIC was proposed by the United States National Highway Traffic Safety Administration and is included in the FMVSS, No. 208. HIC is still the most commonly used criterion for head injury in automotive or sports injury research [6]. HIC is calculated as follows:
$$HIC=\left\{(t_{2}-t_{1}){\left[\frac{1}{t_{2}-t_{1}}\int_{t_{1}}^{t_{2}}a(t)dt\right]}_{\text{max}}^{2.5}\right\}$$
  In the equation, t_1_ and t_2_ are any two arbitrary time points during the acceleration pulse not to lay more than 15 ms, “a” is the acceleration due to gravity, and time is measured in seconds. The resultant acceleration is used for the calculation. Higher HIC values indicate a higher probability of suffering from severe head injuries. The probability of skull fracture associated with an HIC threshold value of 700 for a mid-sized male is 31%.
  Second, for quantitative evaluation of neck injuries, the neck injury criterion, Nij, was developed and used in the FMVSS, No. 208 [7, 8]. This criterion is based on the correlation of data from dummy tests, cadaver tests, and real-world injuries. The Nij criterion was calculated as follows: Nij = (F_z_/F_int_) + (M_y_/M_int_), where Fz represents the axial forces in the upper neck (either tension or compression) and My represents the flexion/extension bending moment at the occipital condyles. F_int_ and M_int_ are critical intercept values used for normalization of differently sized dummies; in the present study, F_int_ was 3,370 in tension and 3,370 in compression, and M_int_ was 155 in flexion and 62 in extension. The Nij includes the four neck injury predictors according to the directions of N_TE_ (tension–extension), N_TF_ (tension–flexion), N_CE_ (compression–extension), and N_CF_ (compression–flexion). The Nij is used to predict upper cervical spine injuries with the intent to assess serious neck injuries with an abbreviated injury scale (AIS) score of 3. An injury threshold value of 1.0 corresponds to a 40% risk of an injury with an AIS score of ≥3 and a 75% risk of an injury with an AIS score of ≥2 [9, 10].

## Results

### Trends in collisions involving vehicles with wheelchair passengers

In the ITARDA database, we identified MVCs involving 15 fatally injured, 119 severely injured, and 870 mildly injured wheelchair vehicle passengers between 2017 and 2019 (Table 1). Among them, seatbelt users accounted for 86.7%, 62.8%, 79.4% of the cases of fatally injured, severely injured, and mildly injured wheelchair user vehicle passengers, respectively. Additionally, between 2020 and 2022, wheelchair user vehicle passengers using seatbelts accounted for 100%, 75.6%, and 77.8% of 10 fatally injured, 82 severely injured, and 501 mildly injured wheelchair user vehicle passengers, respectively. Seatbelt use rates were compared between 2017–2019 and 2020–2022. The differences were not significantly different between the two periods for fatally injured, severely injured, and mildly injured wheelchair users (p-values of 0.23, 0.06, and 0.49, respectively, chi square test).

**Table 1:** Number of rear seat passengers of wheelchair transporting vehicles. Numbers of fatally injured, severely injured, and mildly injured wheelchair vehicle passengers according to seatbelt use are shown (from the ITARDA database).

	**2017–2019**		**2020–2022**	

	**Seatbelt**		**Seatbelt**	
	**+**	**−**	**+**	**−**
Death	13	2	10	0
Severely injured	74	45	62	20
Mildly injured	691	179	390	111

### Sled test using a dummy model

#### Dummy kinematics

**Fig. 2:**
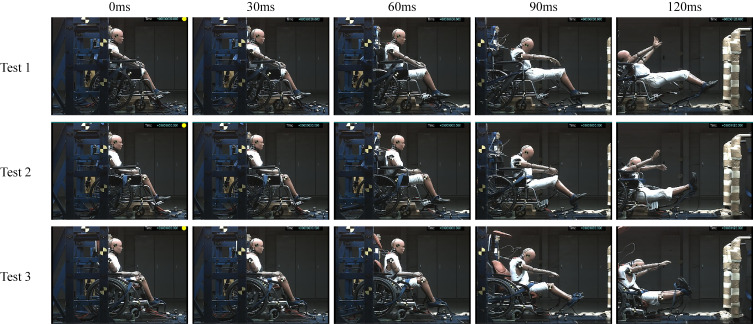
Kinematics of the dummy. Kinematics of the dummy in each test are shown.

In test 1, after the onset of collision, the dummy moved forward and the lap belt compressed the abdomen (the so-called submarine phenomenon), and kept moving forward, with the body slipping forward from the seat. The maximum forward displacement of the pelvis was 661 mm. At approximately 100 ms, the neck of the dummy flexed and the chin impacted with the chest. The head of the dummy then made contact with the backrest of the wheelchair during rebound (Fig. 2). In test 2, after the onset of collision, the dummy moved forward and the lap belt compressed the lumbar region of the dummy with the maximum displacement of the pelvis of 440 mm, then the body of the dummy fell from the seat because the wheelchair seat collapsed (Fig. 2). In test 3, after the onset of the collision, the dummy moved forward and was restricted by both belts with a maximum displacement of the pelvis of 325 mm. The front wheels of the wheelchair were then destroyed.

#### Biomechanical values

**Fig. 3:**
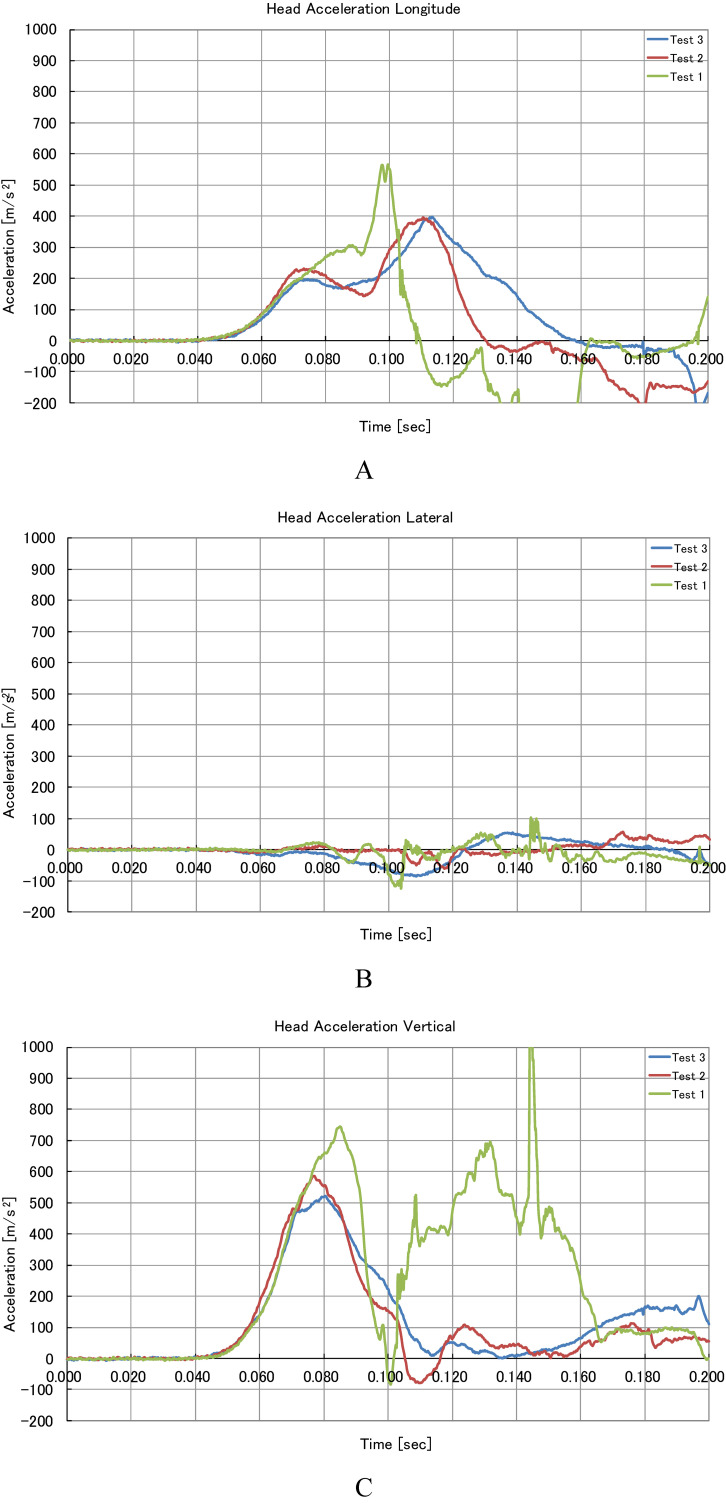
Head accelerations of the dummy. Time courses of the head accelerations in the X (A), Y (B) and Z (C) axes.

**Fig. 4:**
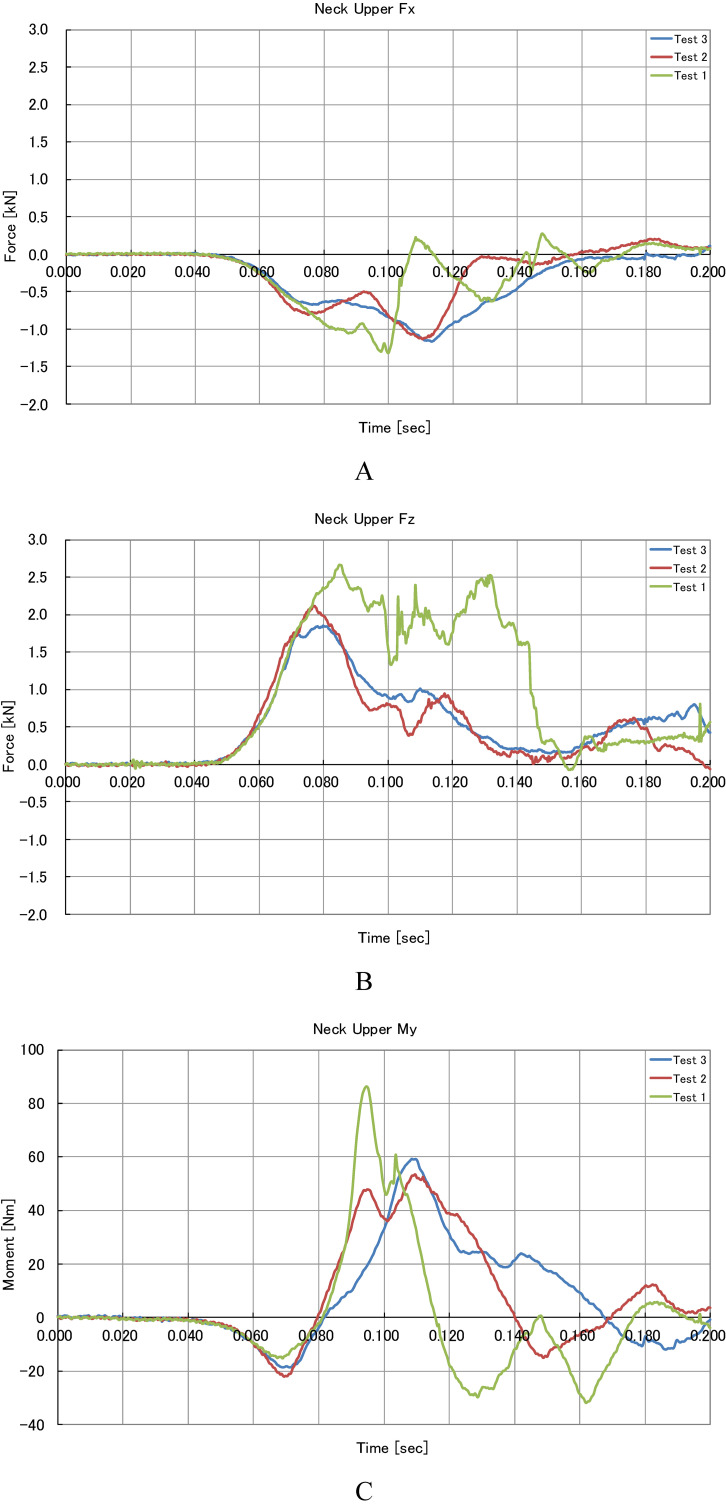
Neck accelerations and the moment of the dummy. Time courses of neck acceleration in the X (A) and Z (B) axes, and moment around the Y axis (C) are shown.

**Fig. 5:**
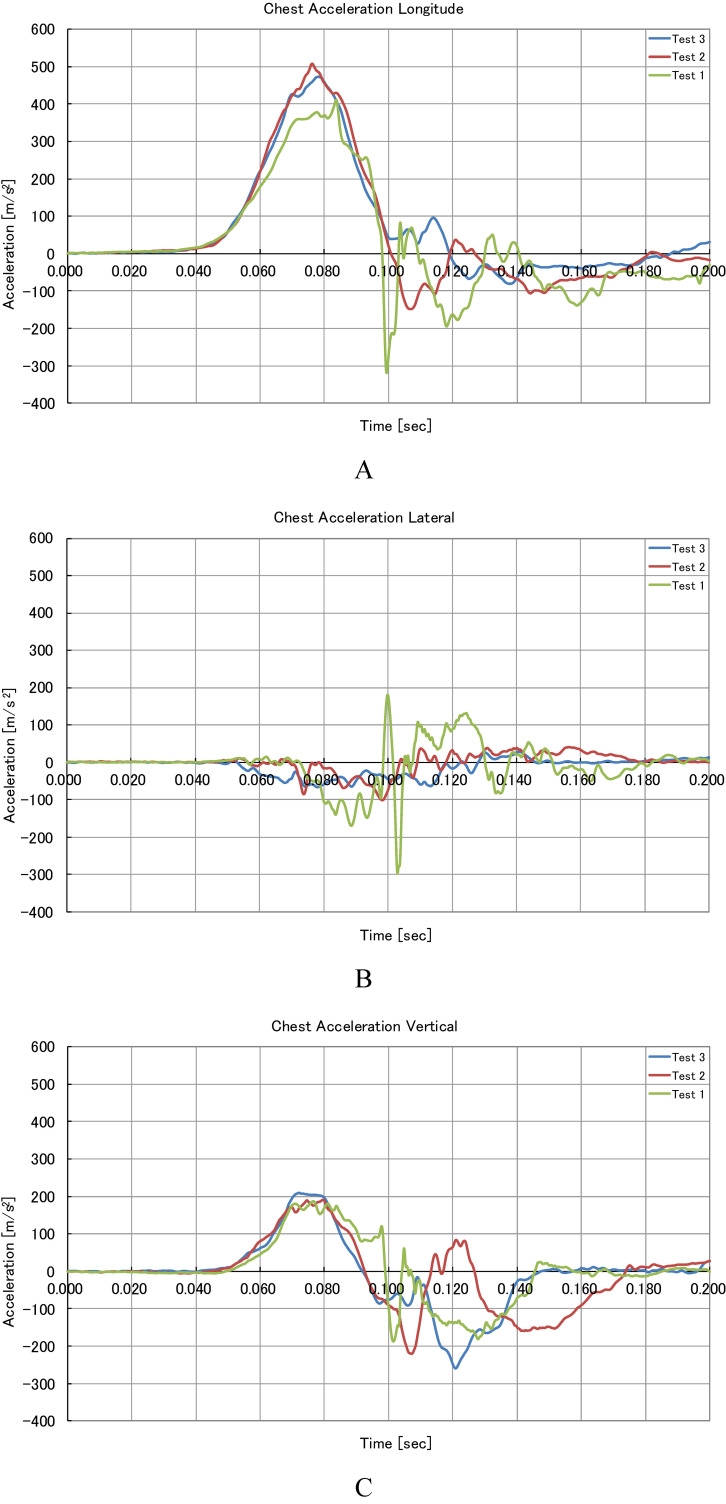
Chest accelerations of the dummy. Time courses of chest acceleration in the X (A), Y (B) and Z (C) axes are shown.

**Fig. 6:**
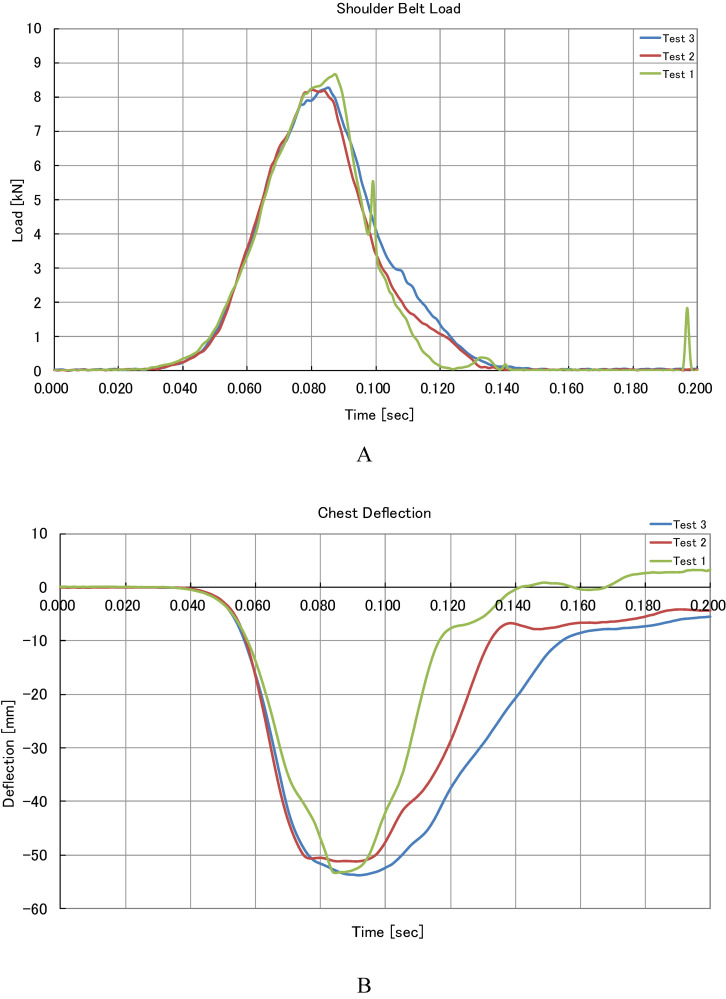
Shoulder belt loads and chest deflection of the dummy. Time courses of shoulder belt load (A) and chest deflection (B) are shown.

Figure 3 shows head acceleration time courses in three axes. Figure 4 shows neck accelerations in the X and Z axes and the moment around the Y axis. Figure 5 shows chest accelerations in three axes. Figure 6 shows the shoulder belt loads and chest deflections. The maximum values of each parameter are summarized in Table 2. High values of head injury criterion (1,274) and neck injury criterion (Nij of 1.05) were observed in test 1, caused by the severe neck flex and head contact in the rebound phase. In all tests, high values of chest deflection (51.3 to 53.8 mm) were found, caused by the high maximum values of shoulder-belt loads (8,660 N at 87.3 ms in test 1, 8,283 N at 80.1 ms in test 2, 8,217 N at 85.2 ms in test 3).

**Table 2:** Summary of the obtained values. The maximum values of each parameter are summarized.

**Variable**	**Test 1**	**Test 2**	**Test 3**
HIC	1,274	390	310
Nij			
N_tf_	1.05	0.52	0.61
N_te_	1.02	0.73	0.68
N_cf_	0	0.04	0
N_ce_	0.35	0.03	0
Max. displacement			
Chest (mm)	380	318	325
Pelvis (mm)	661	440	325
Max. shoulder belt load (N)	8,660	8,217	8,283
Max. chest deflection (mm)	53.3	51.3	53.8

Max: maximum

## Discussion

### Injury mechanisms

A retrospective analysis of the Consumer Product Safety Commission’s National Electric Injury Surveillance System database estimated that approximately 2,294 injuries and deaths occurred between 1991 and 1995 among vehicle occupants seated in wheelchairs as a result of improper securing of the wheelchair [11]. A study involving in-depth interviews of 336 people who regularly used their wheelchair as a seat in vehicles suggested that 28.7% of respondents had suffered from at least one injury while using motor vehicle transportation in the previous 3 years [12]. Schneider et al. examined data regarding 42 wheelchair-seated drivers and passengers from the national MVC database in the United States using the National Automotive Sampling System Crashworthiness Data System [13]. The results revealed that 26 out of 42 occupants in these cases sustained significant injuries and 10 died. Of the 42 occupants, 30 were improperly restrained (e.g., improper positioning of belt restraints on the occupant’s body) [13]. These findings suggest that poor seatbelt fit is the main cause of severe or fatal injuries in wheelchair users involved in vehicle collisions.

In the correct seatbelt position, the shoulder belt lays across the clavicle and sternum and the lap belt interacts with the anterior edge of the ilium under the anterior superior iliac spine. When the seatbelt is appropriately positioned on the dummy, the forward movement of the dummy is kept within an acceptable range. A previous study representing a frontal collision with same velocity as the present study examined the kinematics of the same dummy on the rear seat [14]. The results revealed that maximum forward displacement of the pelvis was 151.1 mm at the collision [14]. Because the dummy was adequately restrained, the value was obviously smaller than that in the present results. The results of the simulation in the current study suggested that the harm caused by the lap belt is a serious issue for wheelchair users. Our initial result suggested that substantial numbers of fatalities and casualties among wheelchair vehicle passengers were seatbelt users. We assume that the seatbelt was not adequately positioned in these real-world cases of fatal collisions involving wheelchair user vehicle passengers. This possibility is in accord with the results of the current sled tests, which demonstrated that an inadequately restrained dummy in which the lap belt was not fitted to the pelvis suffered from high chest load values and the submarine phenomenon.

### Effectiveness of correct seatbelt use

A previous parametric simulation study suggested that wheelchair seated vehicle occupants using a seatbelt with poor belt fit exhibit a higher risk of injury in frontal collisions than those with good belt fit [15]. Senin et al. reported the results of frontal collision sled tests with a similar velocity to that tested in the present study with several wheelchair types, according to the requirements of the ISO [5]. The results suggested that the submarine phenomenon was observed in both steel and aluminum wheelchairs, and the lap belt was positioned on the abdomen of the dummy after the collision in each test [5]. Improper, incomplete, and non-use of seatbelt restraints have been reported to be common causes of serious and fatal injuries to passengers seated in wheelchairs in MVCs [13]. These conditions can lead to ejection from the vehicle, injurious contact of the occupant’s head or chest with the vehicle interior or other occupants, and aggressive load to soft areas of the body, such as the abdomen or neck. To reduce the risk of injury from seatbelt loading in a collision, it is crucial that shoulder and lap belts are correctly positioned across the bony parts of the shoulder and pelvis, respectively, on the wheelchair.

Additionally, because poor seatbelt fit leads to discomfort, wheelchair passengers may hesitate to fasten their seatbelts. Some wheelchair users do not fasten the shoulder belt because it is inappropriately positioned against the neck [1, 2]. According to a survey conducted by a metropolitan transit agency located in the southern United States, 65% of wheelchair passengers failed to use shoulder or lap belts, and failure to use shoulder belts accounted for 98% of these cases [16]. An investigation of 29 adult wheelchair user vehicle passengers suggested that there are several different seatbelt routing configurations: routing over the top of the wheelchair arm support, across the front of the armrests, through an opening under the arm support, and between the arm rest and wheelchair seatback-support [17]. To improve the safety of wheelchair users in motor vehicles, the development of comprehensive restraint systems is needed.

### Shoulder belt force

The present results suggested that excessive shoulder belt loads may play a critical role in severe thoracic injuries among wheelchair-seated passengers, therefore, should be interpreted in the context of existing automotive safety standards. The Ministry of Land, Infrastructure, Transport and Tourism, and the National Agency for Automotive Safety and Victims’ Aid in Japan evaluate the safety of every new car in the country (Japan New Car Assessment Program [JNCAP]). According to the requirements of the JNCAP, when the frontal flat barrier test is performed with a collision velocity of 50 km/h, the obtained values with the Hybrid III AF05 dummy sitting on the rear seat should be as follows: HIC of 700 or less, chest displacement of 42 mm or less, and shoulder belt load of less than 6.0 kN. In the present results, although the values of HIC and chest displacement were within the acceptable range in tests 2 and 3, shoulder belt loads in all tests exceeded the acceptable range. In all tests, because the lap belt did not fit the position of the pelvis, the body was displaced forward, and the higher values of shoulder belt loads resulted in stronger chest deflections. Similar results were observed in a previous study using frontal impact sled tests, in which a collision caused the submarine phenomenon in a dummy in a wheelchair [5]. Additionally, maximum chest deflections of 76.9 mm and 107.3 mm were observed using two types of wheelchairs, which is more than the safe deformation of the thorax itself [5]. For adequately fitting lap belts, concerns may exist regarding the high shoulder belt loads for wheelchair users. Therefore, reduction of shoulder belt loads is also a safety issue. Recently, widespread installation of seatbelt safety systems with a pretensioner and force limiter has dramatically improved safety of vehicle passengers in front seats. According to a previous report, rear seat passengers of vehicles suffer from more serious chest injuries than front seat passengers in MVCs because of the lack of a pretensioner and force limiter [18]. When considering the structure of the rear space of vehicles transporting wheelchairs, we recommend the installation of a force limiter in restraint systems for wheelchair vehicle passengers. Force limiters lessen the risk of upper body injuries by releasing the shoulder belt at forces above a predefined threshold. First, the shoulder belt load should be reduced to less than 6 kN, as required by the JNCAP. Merts et al. reported that chest injuries with an AIS score of 3 did not occur when the shoulder belt load was less than 4 kN [19]. Thus, further efforts to reduce the seatbelt load to this level are required.

### Recommendations for manufacturers

Based on the above biomechanical findings, positioning the lap belt near the lumbar region of the dummy (test 2) resulted in the wheelchair being destroyed and the dummy falling off. Therefore, even when the seatbelt was used correctly, the standard wheelchair did not withstand the forces applied during the collision. In test 1, the dummy moved forward and most of the applied deceleration was received by the dummy via the seatbelt. However, when the dummy was adequately restrained, the applied deceleration was received by the wheelchair and the dummy. Therefore, the sturdiness of the wheelchair against applied forces is an important safety issue. The present results suggest that further studies using biomechanical analyses are required to clarify this issue in more detail.

Generally, to ensure the safety of wheelchair users and nearby passengers, the use of wheelchair tie-downs and correct restraints with lap and shoulder belts are required [16]. In this study, sled tests were performed under the condition that wheelchair was properly tied-down in the vehicle. Therefore, the influence of tie-down system was not examined in the present study. In the United States, four-point, strap type wheelchair tiedowns and lap and shoulder belts are considered to be the primary means for securing wheelchair user passengers. The practice of using tie-downs for wheelchairs was examined using video recording in paratransit vehicles [16]. According to the results, although non-use of tie-downs was observed in only 0.45% of cases, misuse was observed in 20.2% of cases [16]. Therefore, a simple tie-down system consisting of less than four-point straps might helpful for avoiding misuse. However, an effective tie-down system must be in accordance with the estimation of the applied forces to the wheelchair at impact. On the basis of the revised restraint system for wheelchair user passengers, further tie-down methods should be reconsidered. Additionally, because the lack of sufficient help in the field of nursing care causes a physical burden for caregivers, a simple and quick tie-down method is recommended.

Current international standards exist regarding the safety of wheelchair users traveling in vehicles: ISO 10542-1 and 7176-19. In these standards, although the sled test procedure used a 48 km/h, 20 G acceleration pulse and the acceptable displacements of each body region of the dummy on the wheelchair or wheelchair itself were determined, acceptable biomechanical parameters obtained from the sled tests addressed in the FMVSS were not applied [20]. The situation in which a person uses a wheelchair as a seat in a motor vehicle is not comprehensively addressed in the FMVSS. Therefore, we propose to set the new international safety standards for occupant protection systems that provide wheelchair-seated passengers with a similar level of protection as passengers on the vehicle seats. Under current road traffic laws in Japan, because wheelchair user passengers do not sit on the vehicle seat, the legal requirement of seatbelt use is exempted for wheelchair user passengers. Therefore, more guidelines regarding the correct method for restraining wheelchair user passengers in the vehicle are needed.

### Limitations

The current study involved several limitations. First, the number of wheelchair user vehicle passengers involved in collisions was not accurately determined according to the ITARDA database. Although in this database, the term “wheelchair user passengers” could not be used as a search term, we collected cases with the terms “wheelchair transport vehicle” and “rear passenger.” This may have introduced selection bias. However, among the obtained samples, we compared the numbers of injured persons according to the seatbelt use. Therefore, we believe that this issue is unlikely to have influenced the present results. Second, a single test was performed in each condition. Therefore, we could not confirm the reproducibility and variability by the obtained data. However, the dummy used in this study was developed for frontal MVC tests and yielded highly reproducible results. The MVC test for the accreditation to the international standard was performed only once. Third, a dummy with a small stature (AF05) was used in this study. According to the National Health and Nutrition Survey in Japan, the average height of individuals aged 75–79 years old was 163.3 cm in males and 149.8 cm in females, and individuals aged 80 years or older had an average height of 161.1 cm in males and 146.6 cm in females [21]. Therefore, we believe that the Hybrid III AF05 dummy, with a height of 153 cm, was appropriate for the present tests. Fourth, in this study, mechanisms of injuries were determined on the basis of a frontal vehicle collision. Because other modes of collision also occur (e.g., lateral or rear-end collisions), other issues may exist regarding the interactions between wheelchair users and the seatbelt or vehicle interior. A survey of real-world MVCs involving wheelchair user passengers reported that frontal collisions were the most common [2]. Future studies simulating other modes of collision may produce useful results.

Currently, when traveling in motor vehicles, wheelchair users are not able to use vehicle manufacturers’ seatbelt restraint systems. Therefore, wheelchair users are travelling with a greater risk of serious and fatal injuries compared with people seated in vehicle manufacturer-designed seats, which involve federally mandated and regulated occupant-protection systems. Passengers seated in wheelchairs in private vehicles have significantly higher injury rates than those who are transferred to vehicle seats (7.5 vs. 4.4 injuries per 100,000 miles travelled), and a similar pattern has been reported in public vehicles (5.2 vs. 0.6 injuries per 100,000 miles travelled) [22]. The current results provide manufacturers with important and reliable information for the development of effective novel restraint systems for wheelchair user vehicle passengers.

The present study might contribute to the traffic safety if more safer restraint system for wheelchair user vehicle passengers is developed. When the safety of older wheelchair users who receive care services are improved with the present results, both physical and psychological burden of care givers would be reduced. Therefore, the present study might also contribute to the smooth implementation of care services for the elderly.

## Conclusion

Our analysis of data from the ITARDA database in Japan revealed that most fatalities and injuries among wheelchair user passengers in MVCs occurred among passengers who were using a seatbelt.

According to the pilot study combining descriptive accident data analysis with biomechanical experimentation, indications for future design of safety restraint system were provided as follows: both the shoulder and lap belts would be correctly positioned across the bony parts of the shoulder and pelvis, respectively; a force limiter would be installed in the restraint system to reduce shoulder belt loads; a sturdy wheelchair and adequate tie-down system that can resist applied forces would be used.

The current results may provide manufacturers with useful data regarding the development of effective novel restraint systems for wheelchair user vehicle passengers, contributing to a decrease in the number of fatally or severely injured passengers in MVCs.

## Declarations

### Availability of data and materials

Data are available from the corresponding author upon reasonable request.
